# Spatially Consistent High-Resolution Land Surface Temperature Mosaics for Thermophysical Mapping of the Mojave Desert

**DOI:** 10.3390/s19122669

**Published:** 2019-06-13

**Authors:** Scott A. Nowicki, Richard D. Inman, Todd C. Esque, Kenneth E. Nussear, Christopher S. Edwards

**Affiliations:** 1 Department of Earth and Planetary Sciences, University of New Mexico, Albuquerque, NM 87131, USA; 2 Quantum Spatial Inc., Albuquerque, NM 87106, USA; 3 U.S. Geological Survey, Western Ecological Research Center, United States Geologic Survey, Las Vegas Field Station, Henderson, NV 89074-8829, USA; rdinman@usgs.gov (R.D.I.); tesque@usgs.gov (T.C.E.); 4 Department of Geography, University of Nevada Reno, Reno, NV 89557, USA; knussear@unr.edu; 5 Department of Physics and Astronomy, Northern Arizona University, Box 6010, Flagstaff, AZ 86011, USA; Christopher.Edwards@nau.edu

**Keywords:** apparent thermal inertia, Mojave Desert, land surface temperature, LST, ecological modeling

## Abstract

Daytime and nighttime thermal infrared observations acquired by the ASTER and MODIS instruments onboard the NASA Terra spacecraft have produced a dataset that can be used to map thermophysical properties across large regions, which have implications on surface processes, thermal environments and habitat suitability for desert species. ASTER scenes acquired between 2004 and 2012 are combined using new mosaicking and data-fusion techniques to produce a map of daytime and nighttime land surface temperature with coverage exclusive of the effects of clouds and weather. These data are combined with Landsat 7 visible imagery to generate a consistent map of apparent thermal inertia (ATI), which is related to the presence of exposed bedrock, rocks, fine-grained sediments and water on the surface. The resulting datasets are compared to known geomorphic units and surface types to generate an interpreted mechanical composition map of the entire Mojave Desert at 100 m per pixel that is most sensitive to large clast size distinctions in grain size distribution.

## 1. Introduction

### 1.1. Surface Geomorphic and Ecological Mapping

High-resolution climate and ecological modeling in arid regions are aided with an understanding of surface characteristics such as rock exposure, soil induration, texture and mechanical composition at fine-scales because these characteristics affect the micro and macro habitat and ultimately determine the assemblage of plant and animal species that may occur [1,2,3,4]. These properties also directly influence the surface and near-surface thermal environments, which can impact water availability and the survival of many threatened reptile and amphibian species [5,6,7]. Improved habitat models have the potential to influence the development of solar and wind energy resources, as well as military installation expansion and residential development across the southwestern US, since such development will likely impact and fragment the habitats of endangered and threatened animal species [6,7]. Separately, changes in climate may also affect habitats for organisms occupying the desert southwest because many climate models predict drastic changes in the global temperature regime and precipitation patterns [8,9,10]. ATI has been shown to be effective for predicting habitat for several species endemic to the Mojave Desert at course spatial scales (1 km) e.g., references [11,12]; however, assessments of environments at spatial scales relevant to organisms are needed to improve the utility of ecological modeling, especially when the aim is to aid land use management and conservation planning [13,14]. Thus, there is a need to develop high resolution environmental data describing the mechanical composition and thermal characteristics of natural and modified surfaces in the southwestern United States.

Data describing surface conditions in the desert regions of the United States are often limited to geologic, geomorphic and pedologic maps, which are generally only resolved at coarse spatial resolutions greater than 1 km, or are only available for specific areas where detailed ground surveys have been completed. Where surveys have been conducted for larger areas, the methods used are often inconsistent from one survey to another, requiring post-hoc standardization across survey areas and loss of geologic detail [15]. For example, geologic maps of the continental USA, such as the integrated USGS geologic database [16], are mapped at moderately high resolutions (1:500,000 scale, or approximately 500 m), but only include geologic age and general parent material type. While helpful in defining the underlying bedrock, this type of map is not useful for describing surface conditions in terms of biophysical properties available to organisms in the context of ecological modeling. Predictive pedological maps may offer greater detail about the surface characteristics due to the breadth of soil properties commonly included such as soil texture, mineralogy, and organic matter content, and secondary properties such as bulk density, pH, or ion-exchange capacity. The Digital General Soil Map of the United States [17] provides an assessment of these soil characteristics that are relevant to habitat, but is only available at a spatial resolution of approximately 2 km across the continental USA. Survey areas with finer spatial resolution (1:250,000; approximately 250 m) are available for some parts of the continental USA, though are lacking in many areas of the arid southwest. 

### 1.2. Thermophysical Mapping 

**Figure 1:**
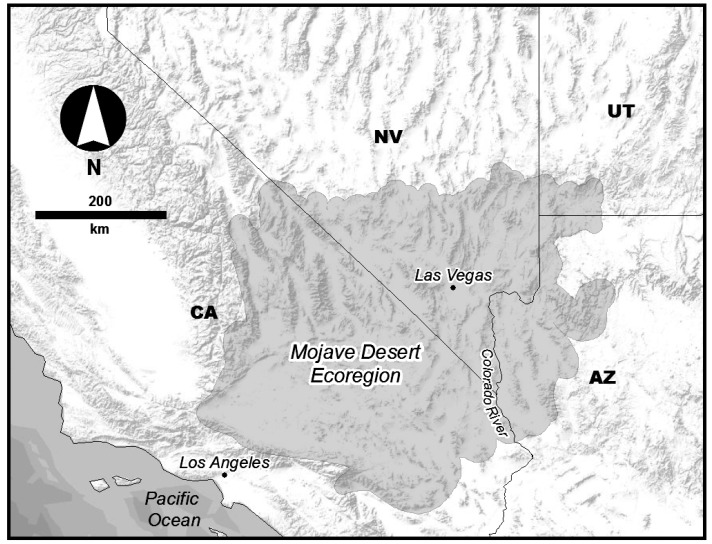
Study area used for thermophysical mapping. The Mojave Basin and Range Level III Ecoregion (Wiken et al., 2011) provides an ideal region for mapping surface thermophysical characteristics due its low vegetation cover, arid conditions, and high prevalence of cloud free days.

Remote sensing datasets provide an opportunity to quantify the areal variation of physical properties of the Earth’s surface using spatially-extensive and consistent methods. This is especially applicable in the Mojave Desert (Figure 1) Ecoregion [18], which is one of the major deserts in the southwestern United States, encompassing portions of southern Nevada, California, Arizona and Utah. The Mojave is a sparsely-vegetated, arid landscape with minimal seasonal cloud cover and precipitation. These conditions make remote sensing-based measurements ideal for differentiating and modeling the physical properties of the region. One useful approach for determining the physical properties of the surface uses visible and thermal infrared (TIR) multispectral remote sensing observations to derive land surface temperature (LST) and albedo that can be used to generate apparent thermal inertia (ATI) [19,20,21,22]. ATI is a relative measure of the thermal conductivity and heat storage of the surface layer, which can be used to characterize the mechanical properties of the upper few centimeters of the surface. These mechanical properties result from the sediment grain-size, soil moisture content, and the presence of crusts and layering [20,21,22,23,24], and help define the suitability for biotic communities. High-resolution environmental data are essential to conservation efforts in many ecosystems, and here we develop and test a method for mapping surface thermophysical properties with Advanced Spaceborne Thermal Emission and Reflectance Radiometer (ASTER), Moderate Resolution Imaging Spectroradiometer (MODIS) and Landsat 7 remote sensing datasets. The resulting data products can be used to interpret surface physical properties appropriate for application to ecological modeling in the Mojave Desert, although these methods are just as applicable to many arid regions around the world. We use the terms high-resolution to describe satellite-based datasets that have ground sample distances of <100 m/pixel, whereas moderate to low resolutions are on the order of hundreds of meters to multiple km per pixel. 

**Figure 2:**
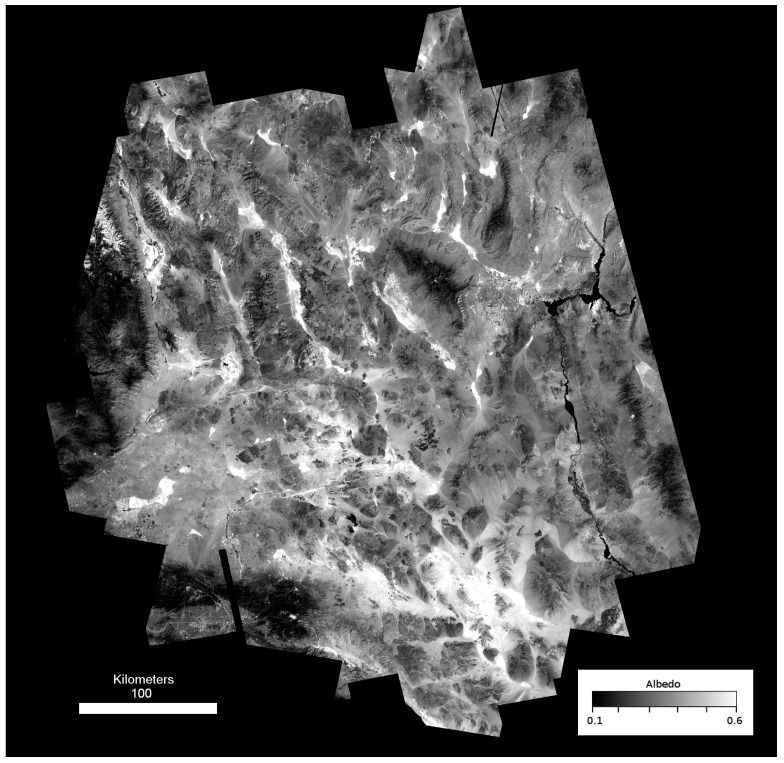
Landsat 7 albedo map for the Mojave compiled using scenes acquired summer 2010.

Two thermophysical properties dominate the surface temperatures of geologic surfaces exposed to daily solar radiation: reflectance and thermal inertia. Reflectance is the ratio of reflected energy to incident solar energy at the given incidence angle, which can be derived from multispectral satellite imagery (Figure 2). Thermal inertia is a property that is best described as the resistance to temperature change caused by time-varying solar insolation [25,26,27,28,29,30], typically observed from satellite-based TIR imagery. An approach that combines thermophysical observations into a consistent and interpretable value is the apparent thermal inertia method [20,22], in which albedo, a measure of the broad-spectrum reflectance, and the difference between daytime and nighttime temperatures are used to map the relative thermal inertia in a remote sensing scene, described as:

ATI = N C (1 − α)/(T_day_ − T_night_) (1)

where α is the albedo, and T is the daytime or nighttime temperature of the surface, N and C are optional scaling factors commonly applied to account for latitude and solar inclination angle, and to normalize ATI to the standard thermal inertia units range for most observations. The latter two parameters are not included in this analysis because spatial corrections for latitude and solar angle are accounted for in the mosaicking methodology presented here. Ideally, the maximum and minimum temperatures for local diurnal cycles are used, but the timing of image acquisition from orbital remote sensing instruments is a function of latitude, which does not always correspond to the daily maximum and minimum (but is consistent for every image). The values produced using this method are dependent upon the surface temperatures from each day and night pair. Since the absolute difference between day and night temperatures is highly dependent upon solar insolation, and varies with season and the local time of the observation, ATI is considered a relative measure of physical properties. When used over a region with a wide range of thermal inertias, the variation in ATI values can be correlated with physical properties that represent interpretable units of sediment grain size, induration, bulk density, and rockiness.

Deriving ATI images with daytime and nighttime LST and daytime reflectivity is straightforward for surfaces with low topography [20,21,22], and when surface materials are homogenous, the interpretation of ATI values can be attributed to an isolated geophysical variable such as soil moisture [21,24]. Surfaces with complex heterogeneity or extreme topography and significant variations in incidence angle can produce ATI values that are not easily interpreted and are not characteristic of the thermophysical properties of the surface. Topographic corrections for slopes have been previously developed [28], but are not able to correct for daytime shadows caused by rugged terrain. Considering the range of elevation, geologic materials, physiographic features, vegetation and climate extremes across the Mojave, measured thermal properties display a wide range in moderate-resolution thermal observations [31]. Heavily vegetated surfaces, bodies of water, and wet soils display surface temperatures that are affected by the thermal conductivity of water and the effects of evapotranspiration [21,23,30]. The focus here is to characterize and map the invariant landscape materials in the Mojave Desert, and therefore some problematic surfaces such as high-slope terrain and variably wet surfaces will only be discussed briefly.

### 1.3. High-Resolution TIR Mosaics

A limitation of currently available high-resolution remote sensing imagery is that the probability of acquiring cloud-free coverage over a large region and narrow time span is low. As a result, regional mapping necessitates the use of scenes acquired from multiple seasons and even years, thereby introducing scene-to-scene variability in mosaics. Efforts to overcome this limitation have resulted in mosaicking techniques for thermal infrared imagery from the ASTER instrument acquired at different dates, and has resulted in data products such as the North American ASTER Land Surface Emissivity Database (NAALSED) [31]. Although the NAALSED dataset includes a spatially-continuous land surface temperature (LST) product, it has not been normalized for seamless thermophysical analyses. Efforts by Scheidt, et al. [32] employed a radiometric normalization method for mosaicking thermal-infrared ASTER radiance images used to map spectral variability over the Gran Desierto, an extensive region of sand in Sonora, Mexico. In both of these efforts, the focus was on the derivation of higher quality emissivity products. Additionally, Scheidt et al. [32] use TIR ASTER radiance imagery to achieve a consistent representation of thermal inertia for White Sands, NM where they were able to isolate and attribute thermal inertia to soil moisture variations through time. However, radiometric balancing was not required to analyze the White Sands dune field because of the small study area, whereas larger areas would require additional image mosaicking steps. Other recent work takes advantage of the temperature trends between subsequent MODIS scenes and estimates changes between them using a spatio-temporal fusion model to derive higher resolution thermal datasets (e.g., Landsat; [33]).

Advances in mosaicking methods have also emerged from the planetary remote sensing community. Data returned from the 2001 Mars Odyssey Thermal Emission Imaging System (THEMIS) [34] also show significant scene-to-scene variability in regional mosaics. A series of THEMIS algorithms were developed to produce a planetary TIR mosaic; however, the use of a running histogram stretch in these algorithms resulted in temperatures that were not quantitatively consistent across multiple scene tracks [35]. These algorithms were designed to minimize heterogeneity in daytime and nighttime temperature images and therefore require an additional step to recalibrate any mosaics produced by them. We describe a toolset of image processing algorithms to produce a quantitatively consistent ASTER TIR imagery and apply it to create an ATI image of the Mojave ecoregion.

## 2. Methods 

### 2.1. Remote Sensing Data

The ASTER instrument on-board the NASA Terra spacecraft collects multispectral imagery at spatial resolutions of 15 m in visible and near-infrared (VNIR), 30 m in shortwave infrared (SWIR) and 90 m in thermal infrared (TIR) wavelengths [36]. TIR data are collected in five distinct bands between 8 and 12 microns and are calibrated to radiance (W/m^2^/sr/micron) that can be separated into a brightness temperature and a five-band emissivity spectrum [37] highlighting areal mineralogic variability. Brightness temperature is calculated as the temperature of a theoretical blackbody emitting the same radiance (Watts/m^2^) as that measured by ASTER over the wavelengths integrated by the five band passes. ASTER is ideal for high spatial resolution mapping, but most locations on the Earth are observed every 16 days at best, and many regions are not regularly observed. MODIS is a set of two identical instruments on board the NASA Earth Observing System (EOS) Terra and Aqua satellites [38] that collect multispectral observations with global coverage twice per day with a viewing swath width of 2330 km. Its detectors measure 1000 m/pixel for thermal infrared (TIR) wavelengths. The Landsat 7 instrument collects six spectral bands with a spatial resolution of 30 m/pixel in the VNIR region (0.45 to 2.35 µm), with an additional band in TIR (10.4–12.5 µm) at 60 m/pixel, and an additional high-resolution VNIR panchromatic band (0.52–0.9 µm) at 15 m/pixel. Landsat 7 orbits in sequence with the Terra satellite with the same 16-day repeat cycle. 

### 2.2. Data Acquisition and Processing Steps

**Figure 3:**
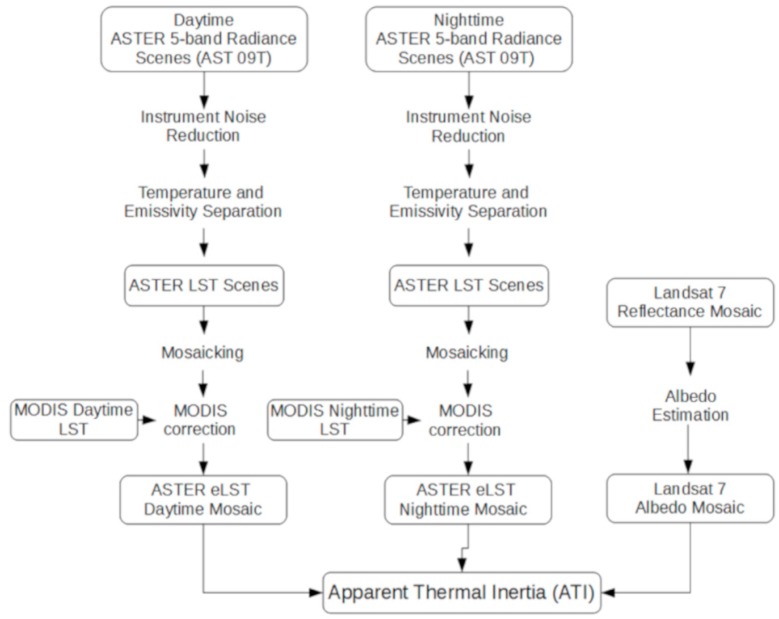
Workflow diagram for the ASTER, MODIS and Landsat-derived ATI map for the Mojave Desert. Closed boxes contain data products, and process steps are listed in the order of processing.

A total of 159 ASTER scenes were compiled to create nighttime and daytime LST datasets, acquired from May 2004 through November 2011 (Table 1). Differences due to long time intervals between images were minimized by leveraging the radiometric consistency and large spatial footprint of MODIS temperature data. A high resolution (90 m/pixel) daytime albedo dataset was generated using a compilation of Landsat 7 scenes acquired between 1 May and 30 September 2010, approximating the core date of 7 June 2010 for the daytime LST. All of the described methods are illustrated in the workflow diagram (Figure 3) which highlights data products and processing steps for the three inputs for the ATI calculation. 

### 2.3. Scene Selection

ASTER and MODIS data products were obtained through the NASA Earth Observing System Data and Information System portal, currently available as the EarthData search portal (http://search.earthdata.nasa.gov). Daytime and nighttime ASTER TIR scenes were obtained as calibrated surface radiance (AST_09T), on which we applied an instrument noise reduction step prior to the emissivity-temperature separation. A total of 85 and 74 scenes were used for each of the daytime and nighttime TIR mosaics, respectively. MODIS daytime and nighttime atmospherically-corrected and calibrated LST and emissivity products (MYD11A1) were used to evaluate the accuracy of the mosaicking algorithms. The composite 2010 Landsat 7 dataset was compiled with Google Earth Engine (http://earthengine.google.org) using the Landsat Ecosystem Disturbance Adaptive Processing System method [39], providing the most consistent and shortest acquisition time span available for albedo reflectance mapping. Narrowband multiband reflectance was used to estimate albedo [40] and resampled at 100 m/pixel, as described in Section 2.5.

ASTER scenes were selected to minimize cloud cover and maximize continuity of scene acquisition, warm season, and projection consistency between scenes. Visual inspection of each scene was necessary to verify image quality because the normal cloud detection methods are not accurate at night due a lack of VNIR observations. Only TIR observations are acquired at night and any clouds can display similar temperatures as the surface, making it difficult to automatically determine cloud cover. However, we identified cloud cover in the nighttime radiance images by the shape and pattern of temperature anomalies in the TIR observations and eliminated cloudy scenes manually. Scenes acquired during warm and dry seasons were selected where possible to obtain the most consistent thermal properties. Additionally, scenes in sequence along a single orbit track are preferred because contiguous observations have virtually no offset in solar conditions. These scenes can be mosaicked into image strips without modification of edge or overlap values, resulting in a seamless mosaic strip in the along-track direction. 

### 2.4. Line- and Row-Correlated Noise Removal

Nighttime ASTER thermal observations with lower surface temperatures have low signal to noise ratios and produce images with characteristically high detector noise that can be seen as image striping. Similar detector noise has been identified and characterized in other multispectral imaging systems and has been known to propagate and intensify with data processing such as emissivity-temperature separation [37]. A post-acquisition technique has been developed to isolate and remove this detector noise, commonly referred to as “plaid”. We use the *deplaid* algorithm, a technique to remove both line (cross-track) and row (along-track) correlated noise from multispectral data sets [41], which has been shown to reduce detector-derived noise and improve the accuracy of compositional analysis of small areas with a very low rate of introducing artifacts and generate more spatially consistent brightness temperatures [42]. The *deplaid* algorithm can be applied to any multispectral dataset where the absolute radiance differences between spectral bands are relatively small, as is the case for multispectral thermal infrared radiance. This is required because the *deplaid* algorithm uses inter-band variation to determine the location and magnitude of line- and row-correlated noise. We modified the *deplaid* algorithm for ASTER imagery by adding an additional step of segmenting each image into horizontal components for redundant noise calculations. In order to remove the detector noise in ASTER-derived temperature imagery, the *deplaid* algorithm is run on multi-band radiance data that can later be separated into temperature and emissivity components. All nighttime scenes were processed using the *deplaid* algorithm on radiometrically-calibrated and atmospherically-corrected AST_09T radiance imagery to generate new five-band radiance data with significantly reduced detector noise. 

### 2.5. Mosaic Steps

**Figure 4:**
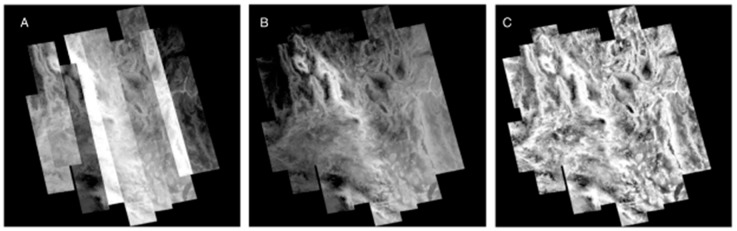
Mosaic steps used in this analysis shown using the nighttime scenes. (**A**) LST temperature orbit tracks are mosaicked without any manipulation of the emissivity-separated temperature, and displayed in relative temperature. (**B**) Each orbit track is adjusted and blended with adjacent tracks to produce a seamless temperature mosaic. (**C**) The seamless mosaic is stretched with a correction filter matched to MODIS LST to produce an image with a consistent contrast across the region.

Following the noise-reduction step on the five-band AST_09T data, surface temperature and emissivity were separated using the ENVI software emissivity normalization process to produce single-band brightness temperature images equivalent to the ASTER land surface temperature (LST) product (AST_08), hereafter referred to as LST. Geographic reprojection of individual scenes and the mosaicking of images in each orbit track into image strips were also performed on LST data using ENVI (Figure 4A). Cross-track mosaicking steps and analyses described below were performed using Davinci, an open source software package developed by the Mars Space Flight Facility at Arizona State University (http://davinci.asu.edu). 

The cross-track mosaicking tools employed here were originally developed for use on THEMIS imagery [35], which have similar characteristics to ASTER imagery. The orbital similarity between the two image sources allows the cross-track mosaicking tools to be used with ASTER imagery when an additional matching algorithm and a MODIS temperature correction filter are applied. The matching algorithm, *level_adjust*, identifies overlapping pixels between adjacent image strips and determines the linear gain and offset correction between the two adjacent strips within the overlapping pixels. This is used to modify pixel values of one image strip to correspond to the overlapping values of the other image strip. The original core strip retains almost all of its original values, while each successive strip has a gain and offset applied, and results in images that are blended to produce consistently scaled temperatures across the mosaic (Figure 4B). 

**Figure 5:**
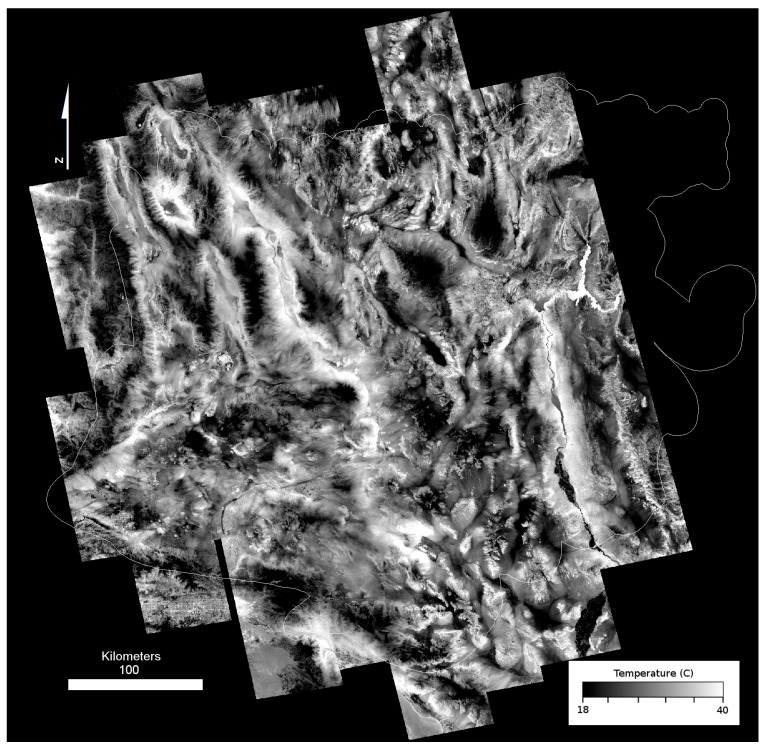
Nighttime ASTER enhanced Land Surface Temperature (eLST) mosaic.

**Figure 6:**
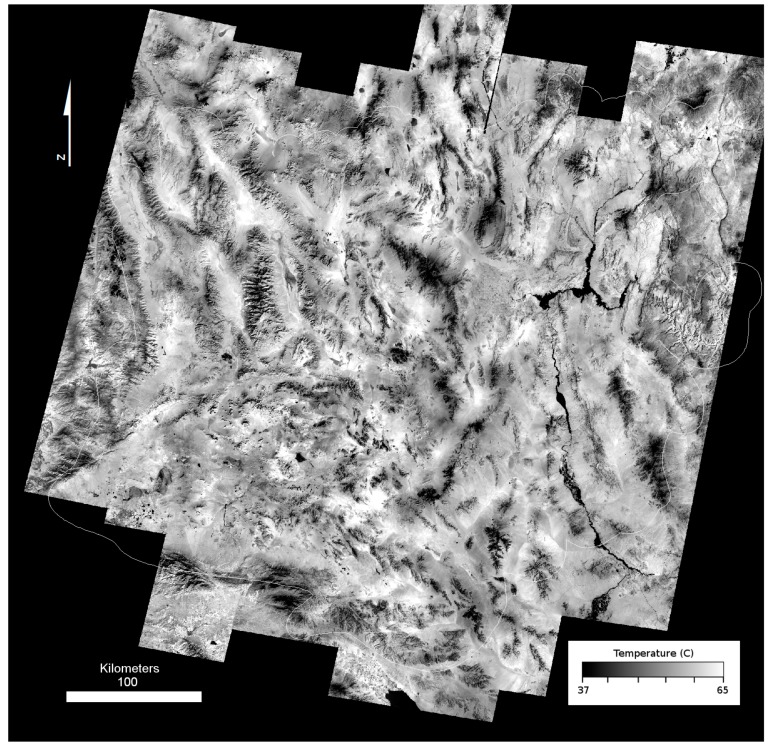
Daytime ASTER enhanced Land Surface Temperature (eLST) mosaic.

While this process does result in a seamless mosaic, it also causes an attenuation of temperature contrast with distance from the core image strip. This attenuation is due to small (<1 pixel) misalignments in projection between images, inconsistent temperature changes due to weather, clouds or seasonal temperature inversions, and statistical error in correlation that propagates from one successive strip to the next [43]. The decrease in contrast in the seamless temperature mosaic becomes more visible with distance from the core image, and suggests a loss in accuracy of temperature. We applied a temperature correction filter method to compensate for this attenuation to minimize the loss of radiometric accuracy and increase contrast across the study area. Our temperature correction filter method used co-registered MODIS thermal observations because MODIS observes the entire Mojave at nearly the same time of day as ASTER. This synchronization enabled the use of a single MODIS scene corresponding to the date of the core ASTER scene (7/1/2010 for nighttime and 6/7/2010 for daytime) to scale the temperature distribution (contrast) across the mosaic (Figure 4C). The core ASTER strip was convolved to MODIS 1 km/pixel resolution and the radiometric offset between ASTER and MODIS was determined and applied to the mosaic. We used a running filter (80 × 20 pixels) to determine the distribution of values in MODIS and applied a regression to the co-registered ASTER mosaic using an 800 × 200 filter. The shape of this filter is designed to retain the more consistent along-track (north-south) temperatures seen in the ASTER imagery. Finally, the entire mosaic was scaled to the original core strip values to produce an “enhanced” land surface temperature (eLST) mosaic for both nighttime (Figure 5) and daytime scenes (Figure 6) in which the pixels corresponding to the core image strip remained unchanged. The ATI datasets were calculated with the eLST datasets and Landsat albedo using methods described above. 

Even when acquired on the same date and time, there are still inherent differences between ASTER and MODIS LST products due to the differences in the temperature retrieval algorithm used by each as well as differences in their respective spatial resolution [44]. However, considering these expected differences, MODIS TIR scenes observed on a single day provide a consistent spatial reference for the mosaic to evaluate systematic errors that may have resulted from seasonal differences, mosaicking steps or the temperature correction filter. Validation of the eLST datasets was performed by convolving these data to the spatial resolution of the MODIS LST scenes and using both a qualitative and statistical test to assess the consistency of our methods across the mosaic.

### 2.6. Field Validation

**Figure 7:**
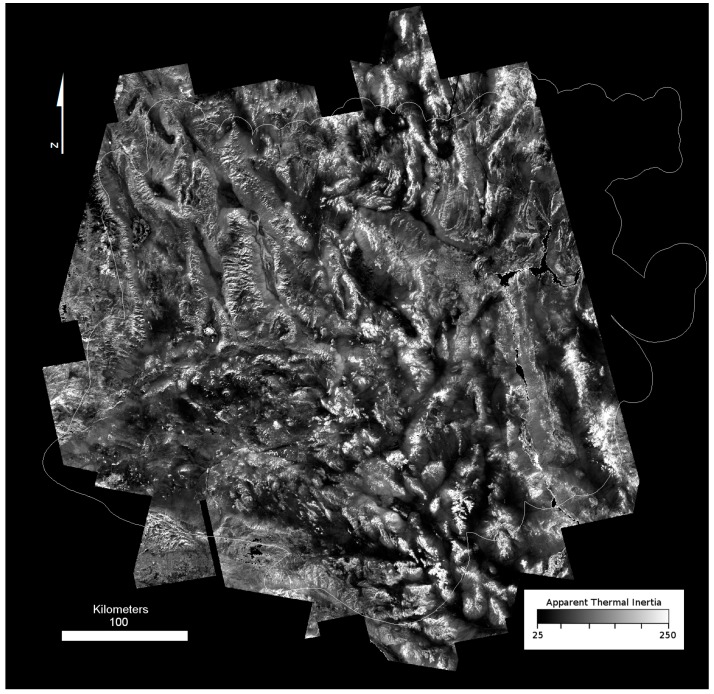
Apparent Thermal Inertia (ATI) of the Mojave. High values represent rockier, more indurated or consistently wet surfaces and lower values represent finer grain size sediments.

**Figure 8:**
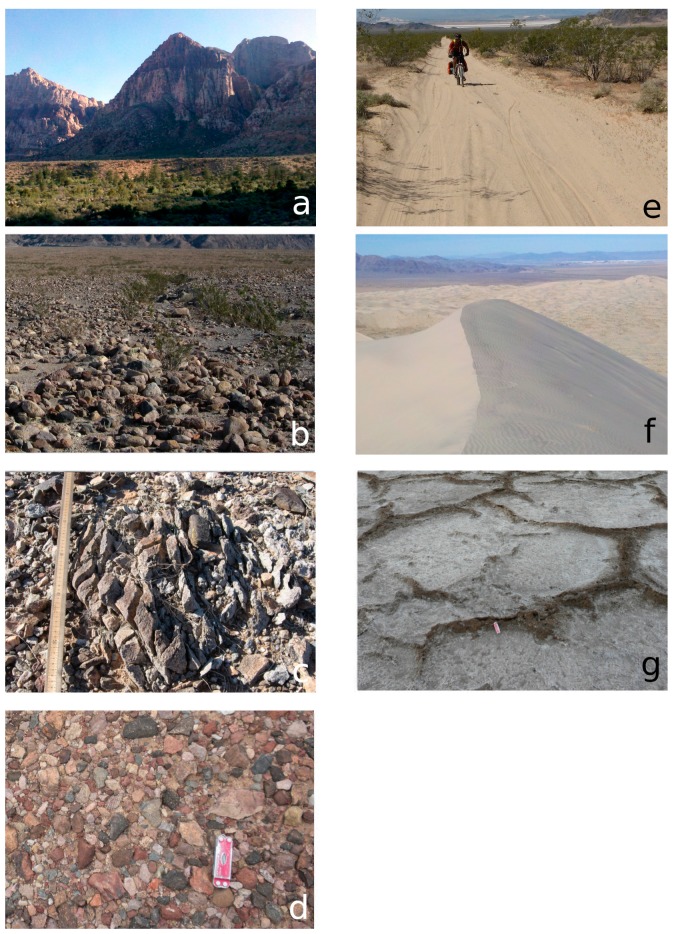
Surface type site examples from the Mojave region: (**a**) bedrock, sandstone cliffs at Red Rock Canyon National Conservation Area; (**b**) blocky, an alluvial fan in northern Death Valley; (**c**) coarse gravel, from near Amboy Crater; (**d**) fine gravel, a desert pavement surface near Tecopa, CA; (**e**) coarse sand, along the Mojave Road in the Mojave National Preserve; (**f**) fine sand, Kelso Dunes; (**g**) silt/clay, the wet playa at Badwater in Death Valley.

Field surveys were conducted at 40 sites using representative surface type classes that could be compared to validate and interpret the range of ATI values seen across the region (Figure 7). Sites were selected from ground-based field observations of mechanical composition, and in all cases the representative surface type corresponded with multiple pixels of consistent ATI values from the dataset. Considering that only a few pixels represents tens of thousands of square meters, surface characteristics were rarely homogeneous within pixels. We took care to select the most consistent and representative field sites for field validation. Surface type classes were based upon visible measurement of the dominant clast size of the coarse portion of the surface. These classes do not take into account the grain size of fine materials (sand, dust, etc.) that are mixed with the course component (rocks, pebbles) in poorly sorted sediments, because these are indistinguishable relative to the larger clasts. Classes included: bedrock dominated by well-cemented sediment, igneous or metamorphic rocks with minimal or no sediment cover (Figure 8a); blocky surfaces where cobble to boulder sized clasts comprise a majority of the surface (Figure 8b); coarse gravel, an unconsolidated mixture of sediment sizes with a component of cobble-sized clasts (Figure 8c); fine gravel, a mixed or homogeneous distribution of grain sizes with the coarse component between 2 mm and 6 cm pebbles (Figure 8d); coarse sand, a poorly-sorted unconsolidated sediment where the majority of the surface is 1–2 mm size clasts (Figure 8e); fine sand, well-sorted wind-borne sand (typically in dunes) (Figure 8f); silt/clay surfaces dominated by clay, silt, and/or evaporate mineral deposits (Figure 8g).

## 3. Results 

**Figure 9:**
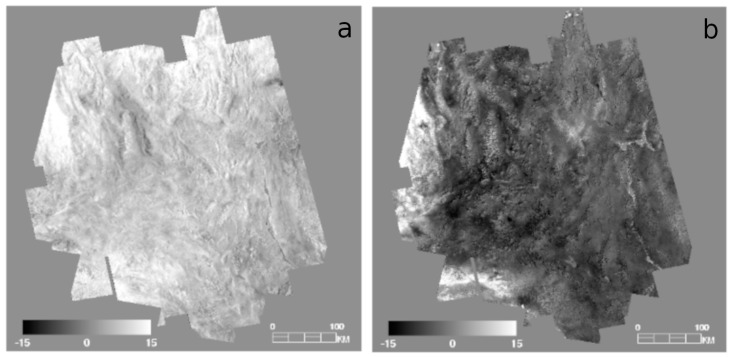
Difference images for MODIS and ASTER eLST in nighttime (**a**) and daytime (**b**), generated by convolving ASTER eLST to 1 km resolution and subtracting it from MODIS LST acquired at the same time as the core image strip date for daytime and nighttime.

**Figure 10:**
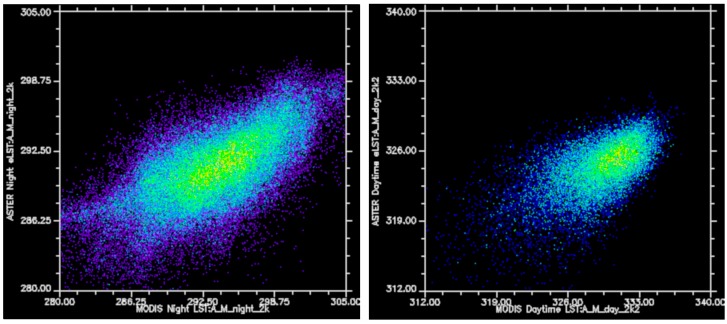
Scatter plots for the spatially-convolved ASTER eLST datasets and MODIS derived LST. Correlation coefficients are 0.861 for nighttime (**left**) and 0.697 for the daytime (**right**) datasets.

Difference images were used to identify discrepancies between the eLST and MODIS LST, and were generated by resampling all datasets to 1 km resolution and subtracting ASTER eLST for day and night to the respective MODIS LST (MYD11A1) dataset. For the nighttime mosaic, 95% of the temperature values lie between −1 and 10 C difference from MODIS, with no distinguishable difference in distribution between the core and the remainder of the mosaic (Figure 9a). For the daytime scene, 95% of the scene lies between −12 and 4 C difference from MODIS (Figure 9b). There is no significant spatial correlation between the daytime and nighttime ASTER/MODIS difference values, and each displays a distinctly different distribution of high and low values. The daytime difference map displays a strong checkered pattern in areas containing the most negative values, most likely due to the effects of shadows and shading within pixels, while the nighttime difference map displays a smoother distribution of values. The difference between the MODIS LST and ASTER eLST can be spatially linked to specific topographic (shadows) and thermophysical (wet/dry) features within the Mojave, and there is little evidence for wide-spread artifacts and negative effects induced during the mosaicking process. Correlation coefficients of the daytime and nighttime eLST datasets to MODIS LST are 0.861 and 0.697, respectively (Figure 10). These positive correlations are comparable to findings from other ASTER/MODIS comparisons [32], considering the number of spatial and temporal discrepancies between the datasets. The correlation for nighttime is higher than daytime due to the lack of shadows at night, and because daytime temperature distribution is highly controlled by season, which varies drastically in ASTER imagery relative to MODIS. Therefore, ASTER night surface temperature distributions are more consistently related to invariant physical surface characteristics than daytime surface temperatures. 

## 4. Interpretations and Discussion

### 4.1. Errors in the Application of ATI

**Figure 11:**
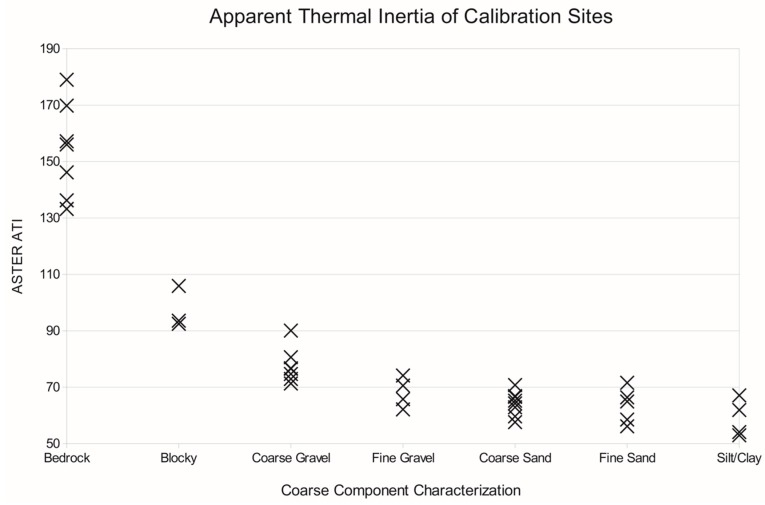
Plot of ATI values for 40 validation sites across the region.

A number of surface substrate types have the potential to display ATI values that are not indicative of the true mechanical composition of the surface (Figure 11). ATI values in these areas suggest that mechanical composition changes diurnally, which is improbable and more likely the result of water or vegetation that buffer diurnal temperature. These surface types included: lakes and rivers, variably wet/dry surfaces, agricultural fields, dense natural vegetation, and snow. Many of the well-defined areas (such as lakes and streams) can be identified and masked prior to deriving the ATI data products. For example, water bodies such as lakes or rivers display low albedo and very high ATI, and can be identified using the criterion albedo <0.07 (which is far darker than even the darkest lava flows). Wet playa conditions can be identified with albedo > 0.3 and ATI > 200, because near-surface water often has a bright layer of silt or salt that is found in most playas after rainstorms. Alternatively, dry playas with bright albedos tend to have much lower ATI values (<70). Some variable wet/dry conditions can often be identified and removed where T_night_ ≥ T_day_, where T_night_ and T_day_ are the nighttime and daytime temperatures, respectively. Vegetation affects the ATI values as a function of cover and density, and the corresponding water content can be mapped using VNIR vegetation indices generated with the Landsat reflectivity mosaic. In previous work, the MODIS normalized difference vegetation index (NDVI) value at which the ATI values show anomalous values occurred around NDVI ≥ 0.2 [29], therefore we mask areas with NDVI above 0.2. Since the presence of elevated soil moisture that occurs following precipitation can result in anomalous ATI values, scene selection process is the best method for reducing ATI variations due to precipitation and soil moisture. The greatest effects of precipitation are found to diminish quickly after storms, such that a day or even hours after a storm event occurs, the temperature change can be undetectable unless soil remains saturated [45]. 

### 4.2. Validation and Interpretation of the ATI Dataset

We find a strong relationship between surface type (e.g., bedrock, coarse gravel, slit/clay, etc.) across the 40 field sites investigated, with ATI values following a decreasing trend from bedrock to silt/clay (Figure 11). In general, higher ATI values correspond to surfaces that have higher block abundances, and larger gravel sizes, or a mixture of these materials. For the Mojave Desert, values >130 are indicative of exposures of bedrock. Values between 110 and 90 were found to represent uniformly large areas dominated by boulders and cobbles; however, this same range can occur with mixtures of rock or blocky materials with finer sand or gravel. Values of ATI below ~90 indicate a maximum grain size of cobble-sized clasts (6 cm). Below values of 75–80, the differences between sand, fine gravel, and silt/clay could not be resolved, and the variation mapped in these classes could be attributed to variations in other characteristics such as slope, soil moisture, induration, surface roughness, vegetation cover, or a number of other possible conditions. These results suggest that across the Mojave Desert, ATI is most sensitive to the presence of cobble-sized clasts or larger. 

For example, we found overlap in ATI values among many of the sandy surface type classes that are the result of several conditions. First, there is a lack of variability in thermal conductivity at finer grain sizes due to the relationship between atmospheric pressure and thermal inertia [46,47]. There may also be characteristics in the surface materials other than dominant grain size not measured here that contribute to altered ATI values. These include sediment induration, variation in the grain size of the fine component and subsurface layering, all of which are important aspects of mechanical composition, yet were not part of our field site characterizations. 

The range of ATI values in our sites containing coarse gravel and blocky surfaces is most likely the result of these characteristics, because the coarse gravel and blocky sites we investigated displayed high variability in sediment provenance and geomorphology. Another condition causing high variability in ATI values is heterogeneity in the spatial distribution of surface materials at the 100-m scale. This is especially noticeable for bedrock surfaces, where large contiguous areas of exposed bedrock are rare, and is complicated by cliffs and other high-angle slopes. Care was taken to choose bedrock field sites that were uniform and horizontal, but contiguous exposed bedrock greater than 100 m in size were rare. At the other end of the ATI scale, the grain sizes most likely produced from a single geologic process (fine gravel, coarse sand, and fine sand) are the most likely to have a homogenous distribution at the multiple-pixel scale.

## 5. Conclusions

With the extensive spatial and temporal ASTER dataset collected over the past two decades, mosaics of nighttime and daytime LST can be produced to model thermophysical properties over large regions that span many orbit tracks. Our data-fusion technique complements a number of different methods that have been used recently to generate mosaics with ASTER [31,32,48] as well as to blend different resolution datasets [33]; however, each have different goals. The methods described here are designed to produce seamless surface temperature mosaics for the express purpose of interpreting thermophysical properties at the highest spatial resolution possible across large areas. Our methods take advantage of tools developed for deriving mosaics from planetary imagers, and enable data fusion between multiple coincident observations from ASTER and MODIS and Landsat 7 instruments to produce apparent thermal inertia. 

The ATI dataset described here represents a modeled value that integrates incident solar radiation, surface reflection and near-surface conduction into a mappable class value. While the high spatial resolution (100 m/pixel) used in this research is appropriate for geomorphology and ecological modeling efforts, it is not high enough to characterize heterogeneous surface textures and thermophysical characteristics at the subpixel level. That is, each value may represent a mixture of materials, and additional insight is required for appropriate interpretation. Values for ATI are interpreted for the Mojave, and are based on a coarse-component of surface materials. The resulting scale can differentiate between unconsolidated fine-grained sediments, blocky and bedrock surfaces, but not between classes of fine grain sizes. Close inspection of the ATI image shows structure across all surface types in the Mojave, suggesting that these methods may be sensitive to surface characteristics that must first be validated by field-based mapping methods. Laboratory or sandbox tests of materials with different mechanical compositions would likely be able to further constrain the applicability of ATI mapping on natural surfaces.

## Figures and Tables

**Table 1:** Acquisition Dates for daytime and nighttime ASTER TIR Mosaics. Each date represents a continuous strip of scenes collected in the same orbit path.

Nighttime ASTER Mosaic Scene			Daytime ASTER Mosaic Scene		
Acquisition Dates	Number of Scenes		Acquisition Dates	Number of Scenes	
11/17/2011	4		10/13/2010	7	
10/17/2011	8		10/11/2010	6	
9/29/2011	7		8/12/2010	7	
8/30/2011	8		6/7/2010	8	Core Strip
7/20/2011	3		9/23/2008	8	
7/2/2011	4		7/19/2008	8	
7/1/2010	8	Core Strip	11/13/2007	8	
4/10/2010	6		8/15/2006	4	
11/17/2009	4		7/13/2005	7	
3/12/2008	3		9/19/2004	7	
3/5/2008	7		6/15/2004	4	
12/2/2006	2				
11/20/2006	6				
8/21/2006	5				
8/14/2006	2				
7/19/2005	8				
	85	Total		74	Total
